# Pulmonary hydatid cyst in a child of 11 years detected by ultrasound lung

**DOI:** 10.11604/pamj.2013.16.137.3539

**Published:** 2013-12-11

**Authors:** Zine el Abidine Benali

**Affiliations:** 1 Department of Anesthesiology & Intensive Care, CHP Eddarak, Berkane, Morocco

**Keywords:** Hydatid cyst, lungs, child

## Images in medicine

Pulmonary hydatid disease is the most common location of hydatid disease of the child. Echinococcus granulosus responsible for this infection is known in the achievement of certain animals such as dogs, man is an accidental intermediate host in parasite's life cycle. The lung is infested after crossing the liver filter, either directly by the lymphatics. The cyst is composed of a germinal membrane, and a pericyst (pulmonary inflammatory response of the host.) It is clinically manifested by cough, chest pain, fever, hemoptysis and hydatid vomica but sometimes asymptomatic incidental finding. Chest radiography is strongly suspected the diagnosis, Partner at thoraco abdominal ultrasound, allows in most cases easy diagnosis. Lung ultrasound is unfortunately largely neglected in this context then it is the key to make this diagnosis without the use of chest CT. Recently Fortia M et al in 2006 have published a new specific sign ultrasound for the wall of hydatid cyst pulmonary: a double layered border in univesicular and double layered internal septum in multivesicular pulmonary echinococcal cysts is a reliable indicator of pulmonary echinococcosis, with a specificity near to 100%. And therefore ultrasonography should not be overlooked for the diagnosis of pulmonary hydatid cysts. To the presence of this sign, is easily eliminates differential diagnosis in the child include: chest neuroblastoma, lung abscess, cystic duplication of the thoracic esophagus, bronchogenic cyst, and pulmonary sequestration. We report a clinical case in a child of 11 years, with notion of contact with dogs, admitted for atypical chest pain with hemodynamic stability in whom a chest X-ray showed a right basal opacity limited, the ECG was normal, thoracic ultrasound showing anechoic image with posterior reinforcement, well limited, with duplication of the wall (germinal membrane hyperechoic and reaction of the host) pathognomonic of hydatid cyst, right basal pulmonary, diameter 8.6/7 cm, type I according to the sonographic classification Gharbi. The heart and abdominal ultrasound (liver, spleen, kidney) without abnormalities, the hydatid serology was negative, the child was operated under general anesthesia, Leaving the hospital six days later without sequelae.

**Figure 1:**
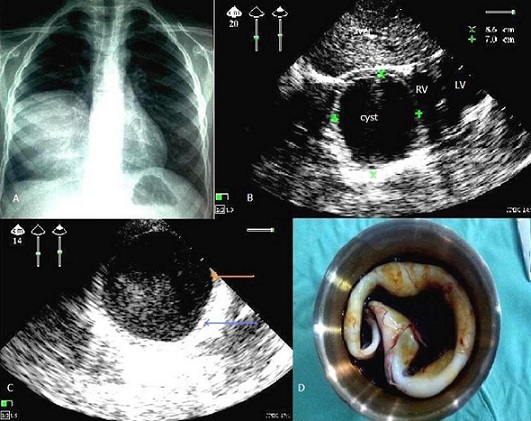
A) chest X-ray (face), showing a right basal opacity, although limited, fluid tonality; B) 2D ultrasound, subcostal through the liver window showing anechoic image with posterior reinforcement, well limited, right basal pulmonary, diameter 8.6/7 cm (RV: right ventricle, LV: left ventricle); C) 2D ultrasound, at right fifth intercostal space on the midclavicular line, showing an anechoic image with duplication of the wall: red arrow for the germinal membrane, blue arrow in relation to the reaction of the host, this is pathognomonic of hydatid cyst; D) operative part of the germinal membrane of the hydatid cyst

